# Impact of different treatment techniques for pediatric Ewing sarcoma of the chest wall: IMRT, 3DCPT, and IMPT with/without beam aperture

**DOI:** 10.1002/acm2.12870

**Published:** 2020-04-08

**Authors:** Zhong Su, Daniel J. Indelicato, Raymond B. Mailhot, Julie A. Bradley

**Affiliations:** ^1^ Department of Radiation Oncology University of Florida Gainesville FL USA; ^2^ University of Florida Health Proton Therapy Institute Jacksonville FL USA

**Keywords:** aperture, Ewing sarcoma, IMPT, IMRT, integral dose, spot size

## Abstract

**Purpose:**

To evaluate the dosimetric differences between photon intensity‐modulated radiation therapy (IMRT) plans, 3D conformal proton therapy (3DCPT), and intensity‐modulated proton therapy (IMPT) plans and to investigate the dosimetric impact of different beam spot size and beam apertures in IMPT for pediatric Ewing sarcoma of the chest wall.

**Methods and Materials:**

Six proton pediatric patients with Ewing sarcoma in the upper, middle, and lower thoracic spine regions as well as upper lumbar spine region were treated with 3DCPT and retrospectively planned with photon IMRT and IMPT nozzles of different beam spot sizes with/without beam apertures. The plan dose distributions were compared both on target conformity and homogeneity, and on organs‐at‐risk (OARs) sparing using QUANTEC metrics of the lung, heart, liver, and kidney. The total integral doses of healthy tissue of all plans were also evaluated.

**Results:**

Target conformity and homogeneity indices are generally better for the IMPT plans with beam aperture. Doses to the lung, heart, and liver for all patients are substantially lower with the 3DPT and IMPT plans than those of IMRT plans. In the IMPT plans with large spot without beam aperture, some OAR doses are higher than those of 3DCPT plans. The integral dose of each photon IMRT plan ranged from 2 to 4.3 times of proton plans.

**Conclusion:**

Compared to IMRT, proton therapy delivers significant lower dose to almost all OARs and much lower healthy tissue integral dose. Compared to 3DCPT, IMPT with small beam spot size or using beam aperture has better dose conformity to the target.

## 1 Introduction

Childhood cancer accounts for less than 1% of all cancers diagnosed each year,^1^ but it is the second leading cause of death in children between ages 1 and 14. In many cases of childhood cancer, a multidiscipline treatment approach that includes surgery, chemotherapy, and radiation therapy is usually adopted to improve patient survival, quality of life, and minimize treatment side effects. Ewing sarcoma is the third most common bone cancer and accounts for about one third of all bone tumors in children.^2^ Ewing sarcoma of the axial skeleton, for example base of skull, chest wall, and pelvis, is frequently treated with radiotherapy that serves as preoperative, definitive, or adjuvant therapy.^3^, ^4^, ^5^, ^6^, ^7^ Due to the proximity of critical organs, radiotherapy is commonly associated with side effects and complications. Ewing sarcoma of the chest wall presents management challenges in the pediatric population affected, the need for aggressive adriamycin‐based chemotherapy, and the presence of critical organs adjacent to the tumor. Treatment with conventional photon‐based radiotherapy is associated with a 26% rate of Grade 3 + toxicity.^8^ Proton therapy — because of the physical properties of protons — has the advantage of sparing healthy tissues and organs‐at‐risk (OARs) and is being used in the management of Ewing sarcoma.^9^, ^10^ Within the last couple of decades, proton therapy availability has increased substantially while the therapy itself has evolved from 3D conformal proton therapy (3DCPT)^11^ using double scattering technique or uniform scanning technique to pencil beam scanning (PBS) using intensity‐modulated proton therapy (IMPT) technique.^12^ In this study, dosimetric comparisons were made between photon intensity‐modulated radiotherapy (IMRT), 3DCPT, and IMPT plans for six pediatric patients with posterior chest wall Ewing sarcomas. Total integrated dose was also calculated for all the plans as a potential indication of secondary cancer possibilities. Within the IMPT plans, proton techniques with and without beam apertures were employed with large and small beam spot sizes to evaluate the dosimetric impact of beam spot size and aperture within the IMPT cohort. This study was a clinical dosimetric study that compared the treatment techniques across widely used radiation modalities and technology representations to provide a realistic assessment of Ewing sarcoma treatment options.

## 2 Materials and Methods

### 2.A Patient information

**Fig. 1:**
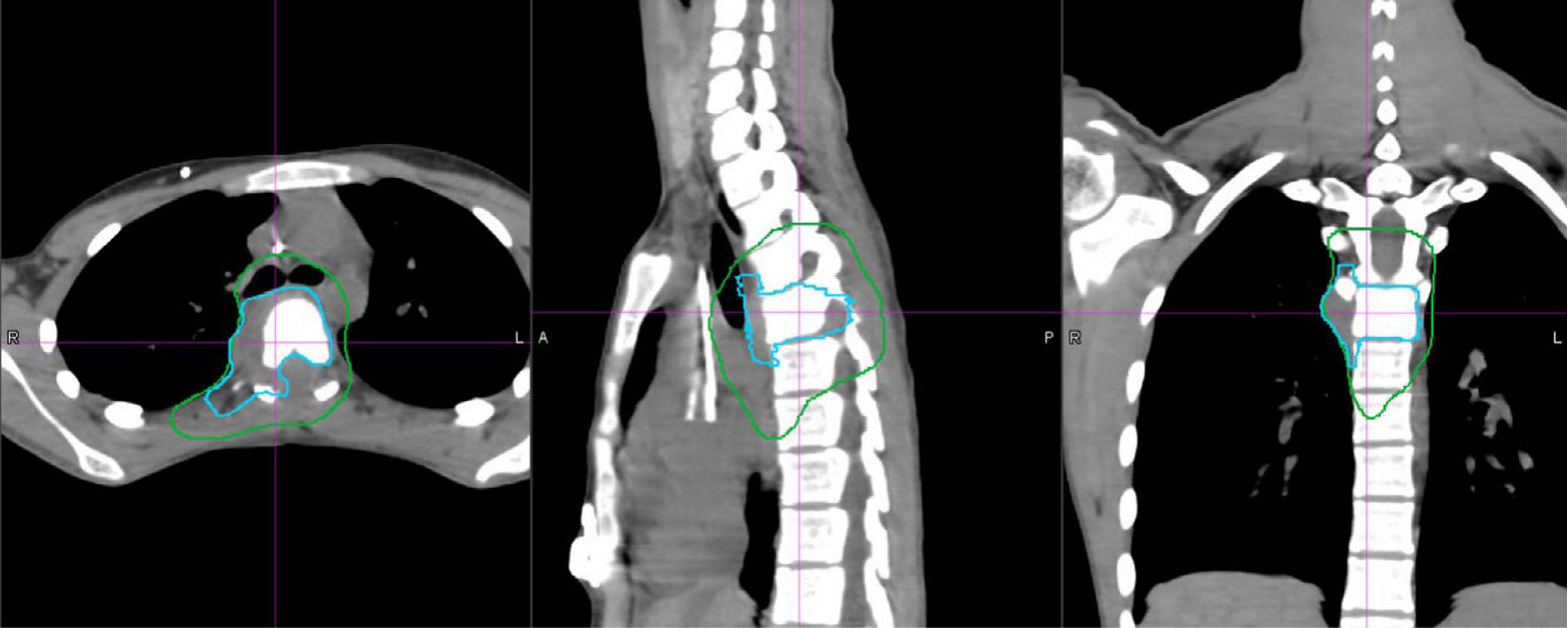
CT axial, sagittal, and coronal views of a patient with chest wall Ewing sarcoma at the region of T5. The green‐colored contour is CTV1 and the blue‐colored contour is CTV2.

Six pediatric Ewing sarcoma patients treated with 3DCPT were selected for this retrospective dosimetric study comparing IMRT, 3DCPT, and IMPT with different beam spot size and with/without beam aperture. Five tumors were unresectable and one was treated postoperatively. These patients were grouped as upper thoracic spine (one patient), middle/lower thoracic spine (three patients), and upper lumbar spine (two patients). Detailed patient information is listed in Table 1. One of the patients who has target volumes at the region of T5 was presented in Fig. 1.

**Table 1:** Chest wall Ewing sarcoma patient characteristics.

Patient	Sex	Age at PT	Therapy Type	Location	CTV1 (cm^3^)	CTV2 (cm^3^)
A	F	12	Definitive	T4‐7	191	35
B	M	3	Post‐Op	T5‐10 + ribs 7	134	58
C	M	3	Definitive	T7‐8 + ribs 7‐9	199	11
D	F	3	Definitive	T9‐10	101	35
E	M	16	Definitive	ribs 11	661	327
F	M	11	Definitive	L2‐3 + ribs 11‐12	919	471

PT, proton therapy.

### 2.B Treatment planning

In this study, clinical target volume 1 (CTV1) received 45 Gy (RBE) in 1.8 Gy (RBE) per fraction for 25 fractions and CTV2 received a sequential boost of 5.4Gy (RBE) in 1.8 Gy (RBE) per fraction for three fractions resulting in a total dose of 50.4 Gy (RBE). The primary criterion of the comparative plan development was equivalent target coverage and the secondary goal was to reduce dose to OAR, including the lung, heart, kidneys, and liver. Integral dose was also tracked to assess second malignancy risk. Standard institutional guidelines on target design, plan optimization, and critical organ tolerance were applied.

The photon IMRT plans were generated using Pinnacle 8.0 m with a step‐and‐shoot technique. The double scattering 3DCPT plans were generated using Eclipse Proton planning system version 11. All the IMPT plans were generated using RayStation 8A.

For 3DCPT planning, the number of beams used for each prescription depended on the target geometry. In general, two or three beams were used. When there were patch fields, the total number of fields increased. For each treatment field, beam distal and proximal margins were customized to take into consideration of the range uncertainties based on CTV. The beam aperture margins were based on planning target volume (PTV) and the beam ranges. The treatment nozzle with beam aperture was extended to about 4 cm from patient or table surface to minimize beam penumbra. Beam compensator smearing margin was customized based on setup uncertainties and potential target motion. Distal blocking was used to reduce dose to the OARs for some of the treatment fields. These double scattering plans were the original clinical plans; the CTV1 coverage was 45 Gy (RBE) covering 99% of volume; and the CTV2 coverage was 5.4 Gy (RBE) covering 99% of volume or 95% of the volume given that 99% was not achievable due to spinal cord dose constraints.

For IMRT planning, five to seven coplanar beams were used for initial target volume and boost target volume for each of the six patients. The total number of segments of each plan was 10 segments multiplying the number of beams. PTV was used for target coverage optimization. The CTV to PTV margins were 5 mm, which were based on interfraction setup uncertainties from x‐ray‐based image guidance, residual interfraction target motion uncertainties, and intrafraction target motion uncertainties. The planning goals were prescription doses covering 95% of the PTV volumes and 99% of the CTV volumes for each of the targets. The OAR dose–volume objectives of the plan optimization were either directly extracted or derived from the QUANTEC metrics of the lung, heart, liver and kidney. When these initial objectives resulted in significant degradation of the target coverage, they were relaxed gradually in an iterative manner during the optimization process until the required target coverage was achieved. To facilitate fair comparisons, the IMRT plans were rescaled based on corresponding CTV coverage of the 3DCPT plans.

For IMPT planning, the beam arrangements were identical among all the four different beam spot and aperture configurations. Two to three beams were used for each of the prescriptions, although the gantry and couch angles may have been different from those of the 3DCPT plans. For the beam computation settings, energy layer spacing was set to “automatic with scale 1” of 80% Bragg peak width; the spot spacing was set to “automatic with scale 0.7” of the spot sigma; for the target margins, there was one layer added on both proximal and distal sides of the target and laterally was set to “automatic with scale 0.7” of the average spot size at the Bragg peak maximum for the highest energy. For the IMPT plan with beam aperture, the PTV to aperture margin was set to 0.7 cm. The robustness settings^13^ were set with 3mm (in all six directions) for positioning uncertainty and 3.5% for range uncertainty. The accurate scenario doses were enabled for all plans. Due to the restriction of minimum energy provided by the proton beam line system, range shifter has to be used for all the beams to provide the appropriate coverage for the shallower portions of these tumors. Proton multiple Coulomb scattering inside the range shifter resulted in the beam spot sigma increase after exiting range shifter. To avoid further beam penumbra degradation, the snout positions were extended to the patient (couch) surface with about 4 cm clearance.^14^ Previous studies indicate that when range shifter was used, a Monte Carlo‐based dose optimization and calculation engine had to be used to ensure accurate dose estimation of the target and normal tissues.^15^, ^16^, ^17^ Therefore, all the IMPT plans generated in this study were optimized using the RayStation Monte Carlo dose engine. Monte Carlo dose calculation algorithm is basically a simulation of proton interactions with patient tissues and deposition of energy along the way. It uses random number to sample the probability density distributions of the physical interaction cross sections for each proton interaction. A properly commissioned Monte Carlo algorithm calculated dose to patient converges to the actual dose to the patient within predefined uncertainty level, which is related to the number of particle histories used in the dose calculations. Since the actual physical interactions in tissues with different compositions and densities were followed, dose in heterogeneous tissues, as in the chest wall Ewing sarcoma patient, can be calculated accurately by Monte Carlo dose algorithm. In contrast, pencil beam algorithm used in 3DCPT is an equation‐based analytical dose algorithm. It is insensitive to tissue heterogeneity perpendicular to the axis of each pencil beam and can present inaccurate dose to some tissues if there is a complex interface between tissues with different densities and compositions. For the Monte Carlo dose calculations in this study, the ion per spot was set to 50000 and the final dose uncertainty was set to 0.5%. Single‐field uniform dose (SFUD) optimization was employed to make each treatment field cover the entire target. The SFUD optimization resulted in a robust dose distribution with respect to setup and range uncertainties. The CTV coverages were rescaled based on corresponding CTV coverage of the 3DCPT plans.

### 2.C Proton machines nozzles and apertures

For the IBA proton therapy systems, there are two types of treatment nozzles designed for IMPT treatment deliveries. The universal nozzle has limited beam vacuum space inside the nozzle that leads to a large beam spot size (in air) at the isocenter (1 sigma from 5 mm to 12 mm for energy from 100 MeV to 227 MeV); the dedicated nozzle has beam vacuum space that extends to the end of the nozzle.^18^ The beam spot size (in air) at the isocenter is smaller than those of the universal nozzle (1 sigma from 3 mm to 7 mm for energy from 70 MeV to 227 MeV). Smaller beam spot size leads to smaller penumbra at the edge of treatment field, therefore, proton plans with dedicated nozzle can potentially reduce dose to the OARs surrounding the target.^19^ For treating the shallower portion of the targets, even though the proton scatter inside the patient is not dominant, the penumbra increases due to a lower proton energy compared to those of deeper targets. Thus, to sharpen the beam penumbra to reduce dose to the adjacent OARs, beam aperture was proposed for IMPT treatment.^20^, ^21^, ^22^, ^23^ In this study, 6‐cm thick brass aperture was used for the nozzle with aperture IMPT plans. For each of these fields, aperture opening was custom fit to that beam‐eye‐view PTV with 0.7 cm treatment lateral margin.

At our institution, both a universal nozzle and a dedicated nozzle are currently in clinical use. Also, aperture‐based IMPT is in the process of clinical validation. Thus, IMPT plans with universal nozzle (IMPT_UN), with dedicated nozzle (IMPT_DN), and both with apertures (IMPT_UN_A and IMPR_DN_A) were optimized in this study for clinical comparisons.

### 2.D Dosimetric comparisons

Since all plans for the same patient were rescaled based on 3DCPT, to evaluate target dose coverage, conformity index (Equation 1 and uniformity index (equation 2) were obtained for each of the treatment plans. To facilitate the OAR dosimetric comparisons among different subsets, dose–volume points were extracted from each treatment plan based on QUANTEC recommendations: for the lung, mean dose and relative volume at 20 Gy^24^; for the heart, mean dose and relative volume at 25 Gy^25^; for each kidney and liver, mean dose was obtained.^26^, ^27^ Normal tissue integrated doses, defined as the energy deposited in the patient body excluding CTV1, were obtained for all plans. The normal tissue integrated dose is highly correlated with the probabilities of secondary cancer incidence; for the pediatric patient population, it is important for plan evaluation. The integral dose was calculated through the total volume of the patient body excluding CTV1 multiplying the average dose (in unit of Gy (RBE)) to this volume (in unit of 1000 cm^3^).

(1) $$\text{Conformity}\;\text{Index}:CI=\frac{\mathrm{TV}}{TV100}\times \frac{V100}{TV100}$$

where TV is the target volume, TV100 is the target volume covered by the prescription dose, and V100 is the total patient volume covered by the prescription dose. Note that the defined conformity index value decreases as the treatment plan becomes more conformal. The lowest conformity index is 1, which represents a plan with perfect conformity.

(2) $$\text{Homogeneity}\;\text{Index}\;HI=\frac{D5\%-D95\%}{\mathrm{Dp}}\times 100\%$$

where, D5% is the minimum dose received by 5% volume of the target, D95% is the minimum dose received by 95% volume of the target, and Dp is the prescription dose.

## 3 Results

### 3.A Target Coverage

The 3DCPT plans had initial 45 Gy (RBE) covering 99% of CTV1. For patient A, B, and C, the boost 5.4 Gy (RBE) covered 95% of CTV2; for patient D, E, and F, the boost 5.4 Gy (RBE) covered 99% of CTV2. All the IMRT and PBS plans were scaled to the corresponding patient 3DCPT plan coverage. Table 2 listed the conformity indices and homogeneity indices of all the treatment plans.

**Table 2:** The conformity indices and homogeneity indices for each of the treatment techniques.

Patient	Indices	IMRT	3DCPT	IMPT UN	IMPT DN	IMPT UN_A	IMPT DN_A
A	CI	6.84	9.70	4.7	5.15	4.31	3.72
	HI	4.7%	15.0%	7.40%	8.1%	7.1%	6.6%
B	CI	4.10	8.72	4.84	4.08	4.22	2.87
	HI	12.5%	9.5%	8.6%	7.4%	8.2%	6.1%
C	CI	11.62	21.16	7.62	8.74	5.57	4.75
	HI	8.0%	8.0%	9.2%	8.0%	8.5%	8.3%
D	CI	3.85	5.44	4.19	3.44	2.76	3.44
	HI	6.8%	7.9%	9.4%	5.2%	6.5%	5.2%
E	CI	2.93	2.82	1.85	1.89	1.60	1.56
	HI	6.7%	6.8%	5.4%	4.5%	4.6%	4.0%
F	CI	2.47	3.20	1.62	1.54	1.54	1.45
	HI	12.5%	9.0%	4.8%	4.5%	4.9%	4.3%

Note

that the conformity index increases as the plan becomes less conformal.

CI, conformity index; HI, homogeneity index; 3DCPT, three‐dimensional conformal proton therapy; IMPT, intensity‐modulated proton therapy; UN, universal nozzle; DN, dedicated nozzle; UN_A, universal nozzle with aperture; DN_A, dedicated nozzle with aperture.

### 3.B OAR Doses

The dose–volume results of all the plans were presented in Table 3. For lung dose comparison, patients C and D’s IMRT plans’ V20 values exceeded the threshold value of 25%. Patient C’s 3DCPT and universal nozzle IMPT plans V20 value also exceeded the threshold. For patient A, B, C, and E, all the proton plans had mean dose and V20 below the threshold values. In general, IMPT with apertures resulted in lower lung dose than IMRT, 3DCPT, and IMPT without apertures.

**Table 3:** Lung, heart, and kidneys dose volume parameters of IMRT, 3DCPT, and IMPT plans.

Patient	OAR	Dose–volume Parameter	Goal	IMRT	3DCPT	IMPT	IMPT	IMPT	IMPT
						UN	DN	UN_A	DN_A
**A**	Lung	Mean Dose (Gy)	<20 Gy	10.6	5.3	6	5	4.7	4.3
		V20Gy	<25, 37%	18.5%	10.9%	11.7%	9.8%	9.1%	8.3%
	Heart	V25Gy	<10%	18.70%	8.10%	8.20%	7.10%	6.50%	5.8%
		Mean Dose (Gy)	<26Gy	11.5	4.6	5.1	4.3	4	3.6
**B**	Lung	Mean Dose (Gy)	<20 Gy	13.3	9	10.5	8.8	9.2	7.5
		V20Gy	<25, 37%	22.7%	19.7%	21.9%	17.9%	19.4%	15.2%
	Heart	V25Gy	<10%	53.6%	0.1%	1.2%	0.9%	0.8%	0.4%
		Mean Dose (Gy)	<26Gy	26.7	0.4	2	1.6	1.7	1.3
	Left Kidney	Mean Dose (Gy)	<18 Gy	4.5	0	0.7	0.3	0.4	0.3
**C**	Lung	Mean Dose (Gy)	<20 Gy	18.1	13.3	12.8	12.2	11.2	10.6
		V20Gy	<25, 37%	32.3%	28.1%	26%	24.4%	23.1%	20.5%
	Heart	V25Gy	<10%	22.7%	1.7%	0.6%	1.2%	0.3%	0.1%
		Mean Dose (Gy)	<26Gy	16.8	1.3	1.4	1.8	1.1	0.9
	Left Kidney	Mean Dose (Gy)	<18 Gy	4.2	0	0.5	0.3	0.4	0.3
	Right Kidney	Mean Dose (Gy)	<18 Gy	1.3	0	0.6	0.3	0.4	0.3
**D**	Lung	Mean Dose (Gy)	<20 Gy	17.5	11.7	12.3	11.4	11.3	11.4
		V20Gy	<25, 37%	35.2%	23.2%	22.8%	21.8%	21.7%	21.8%
	Heart	V25Gy	<10%	58.7%	3.7%	7.7%	6.2%	4.9%	6.2%
		Mean Dose (Gy)	<26Gy	27.8	3.4	5.6	4.4	4.1	4.4
	Left Kidney	Mean Dose (Gy)	<18 Gy	8.9	8.3	9.1	5.5	7.1	5.5
	Right Kidney	Mean Dose (Gy)	<18 Gy	3.6	4.6	6.4	3.8	4.3	3.8
**E**	Lung	Mean Dose (Gy)	<20 Gy	7.1	4	3.1	2.7	2.5	2.1
		V20Gy	<25, 37%	11.9%	8.3%	6.3%	5.4%	5.3%	4.3%
	Heart	V25Gy	<10%	0.1%	0%	0%	0%	0%	0%
		Mean Dose (Gy)	<26Gy	5.3	0	0.1	0	0	0
	Left Kidney	Mean Dose (Gy)	<18 Gy	13.1	1.6	1.4	0.7	1.1	0.6
	Right Kidney	Mean Dose (Gy)	<18 Gy	33.6	17.2	13	11.4	11.8	9.9
**F**	Left Kidney	Mean Dose (Gy)	<18 Gy	39.4	41.3	25.1	24.4	25.1	22.2
	Right Kidney	Mean Dose (Gy)	<18 Gy	18.8	10.4	8.4	6.5	8	5.9

3DCPT, three‐dimensional conformal proton therapy; IMPT, intensity‐modulated proton therapy; UN, universal nozzle; DN, dedicated nozzle; UN_A, universal nozzle with aperture; DN_A, dedicated nozzle with aperture.

For heart dose, patient A, B, C and D IMRT plans had either mean dose or V25 Gy(RBE) exceeded the threshold values. All proton plans had values below the thresholds. For patient D, 3DCPT plan rendered the lowest dose volume parameter values compared to those of IMPT plans.

For kidney dose volume parameter values, patients B, C, and D had all values below the threshold values for both IMRT and proton plans. For the right kidneys of patient E and F, proton plans reduced the dose to kidney below threshold value. For patient F, even though IMPT plans reduced the left kidney dose, the kidney mean doses were still higher than the threshold values.

Liver dose in all of the plans was below the threshold value of 31 Gy. However, the proton plans’ dose to the liver was substantially lower than those of IMRT plans. For IMRT plans, mean liver dose ranged from 11.5 Gy to 20.9 Gy. However, mean liver dose of the proton plans had a minimum value of 0.1 Gy and a maximum value of 4.6 Gy.

Table 4 shows the integrated doses of IMRT, 3DCPT, and IMPT plans. The first observation is that the integrated dose is proportional to the CTV1 volume. 3DCPT plans can reduce the integrated dose to half or one third of those of IMRT plans. Integrated doses of IMPT plans without apertures may or may not be lower than those of 3DCPT plans. However, IMPT plans with apertures have the integrated dose lower than those of 3DCPT plans and it can be as low as one quarter of those of IMRT plans for some patients.

**Table 4:** Integrated dose of IMRT, 3DCPT, and IMPT plans.

Patient	CTV1	IMRT	3DCPT	IMPT UN	IMPT DN	IMPT UN_A	IMPT DN_A
	(cm^3^)	(J)	(J)	(J)	(J)	(J)	(J)
**A**	190.5	64.6	32.7	36.1	31	28.9	27.2
**B**	133.9	46.8	16.1	18.4	16	16	14.1
**C**	199.3	60.5	27.6	29.3	27	26.3	24.6
**D**	101.4	39.9	17.1	18.6	15.5	15.6	15.5
**E**	661.1	182.3	69.9	56	47.6	48.8	42.7
**F**	919.1	147.3	63.6	45.9	44.3	43.6	38.2

3DCPT, three‐dimensional conformal proton therapy; IMPT, intensity‐modulated proton therapy; UN: universal nozzle; DN, dedicated nozzle; UN_A, universal nozzle with aperture; DN_A, dedicated nozzle with aperture.

## 4 Discussion

Radiation therapy is an essential part of management of Ewing sarcoma in the chest wall, either as a definitive or an adjuvant therapy. In the early years of radiotherapy, three‐dimensional conformal photon therapy (3DCRT)^3^, ^4^, ^5^, ^6^, ^7^ was the main radiation treatment technique. But in the last two decades, IMRT has largely replaced 3DCRT as the main radiation therapy planning and delivery technique.^28^, ^29^ Due to the proximity of the lung and heart to the chest wall tumors as well as the physical properties of high‐energy photon beams, multiple beams have to be used and each beam deposits radiation dose in normal tissues before and after the tumors. Toxicities related to radiation therapy include cardiotoxicities, constrictive pericarditis, scoliosis, and pneumonitis. In our prior institutional retrospective study, we showed that even though the modern systemic therapy improved patient cause‐specific survival rates substantially, 26% of the patients experienced Grade 3 + toxicity.^8^, ^30^ Furthermore, secondary malignancy is also a concern of radiotherapy, especially for the pediatric patient population. Several early studies^31^, ^32^, ^33^, ^34^ reported various number of radiation‐induced secondary malignancy with the number as high as 35% at 10 yr. A more recent review by Caruso et al. showed cumulative incidence of secondary malignant neoplasm ranged from 0.9% to 8.4% and 10.1% to 20.5% at 5 and 30 yr, respectively, after initial diagnosis and treatment of Ewing sarcoma.^35^ Therefore, dose reduction to the healthy tissues and OARs surrounding the tumors using proton therapy is necessary. It can reduce radiation treatment‐related toxicities as well as potentially reduce secondary malignancies related to radiation treatment. Paganetti et al. established a lifetime attributable risks model of developing secondary malignancy from radiotherapy treatment.^36^ In all the Ewing sarcoma cases in his study, the calculated integral dose from proton therapy is about a factor of 2 lower than that from IMRT, which is in a good agreement with the results from this study. Their model showed that absolute risk of secondary malignancy can be up to a factor of 11 between proton therapy and IMRT due to the nonlinear dose–response curves predicted by the model.

In this study, for IMRT, 3DCPT, and IMPT_UN plans, there were radiation modality comparisons as well as forward planning vs inversely optimized plan comparisons. For targets, there were no obvious trends in conformity and homogeneity indices. However, for OARs, proton plans did show systematic lower doses than those of IMRT, except for the mean dose of the left kidney of patient F and the slightly higher mean dose of the 3DCPT. Between 3DCPT and IMPT_UN, the doses were comparable and there was no obvious trend. This was likely due to the trade‐off between sharper beam penumbra of the 3DCPT beams and inverse optimization of OAR dose in the IMPT_UN plans. For integral dose, 3DCPT and IMPT_UN had substantially lower values than those of IMRT. However, four of six patients of IMPT_UN had slightly higher integral dose than those of 3DCPT. One of the possible reasons is that 3DCPT utilized beam apertures that sharpened the penumbra of the proton beam compared to IMPT_UN, which did not use any beam apertures^37^ and had relatively large beam spots.

Beam apertures were used for 3DCPT, IMPT_UN_A, and IMPT_DN_A plans. Almost all IMPT plans showed improved CI and HI values compared to 3DCPT plans due to their ability to inversely optimize the spot locations and weights. For the lung, IMPT plans showed lower mean dose and lower volume percentage at 20 Gy; for the heart, four of five patients had lower mean dose and lower volume percentage at 25 Gy in the IMPT plans; for the kidney, two of five patients had near zero dose to the kidneys, and for the other three patients, the IMPT plans all had lower doses than those of 3DCPT. For the liver, two of five patients had near zero doses in all plans. The other three patients had comparable doses between 3DCPT and IMPT plans. Since all the three techniques compared here had apertures in their plan, beam spot size and ability to inversely optimize dose distribution were the two important factors. Therefore, IMPT (large or small spots) with aperture would generate plans had better OAR sparing because of the ability to inverse optimize the dose distribution; IMPT_DN_A plans would be better than 3DCPT and IMPT_UN_A plans in both target conformity and OAR sparing. Similarly, for integral dose, IMPT_DN_A plans were lower than IMPT_UN_A plans that were lower than 3DCPT plans. For patients E and F, there was about 40% reduction of integral dose of IMPT_DN_A compared to 3DCPT.

When comparing all the IMPT plans, the CI and HI had comparable values while IMPT_DN_A plans were always the best of all four types of plans. Similarly, for OARs, IMPT_DN_A showed the lowest doses and integral doses of all four plans. For the same type of nozzle, the plans with apertures always had slightly lower OAR doses and integral doses. Between plans of dedicated nozzle without aperture and universal nozzle with aperture, the OAR doses and integral doses were comparable but without obvious trend.

Among the comparisons of all modalities and techniques in this study, IMRT had the most occurrences of exceeding QUANTEC dose–volume points. Two of six patients had lung dose that exceeded the tolerances; four of six patients had heart dose that exceeded the tolerances; two of six patients had kidney dose that exceeded the tolerances. For patients B and D, the IMRT plan exceeded the tolerances in both mean dose and V25Gy, which could lead to cardiotoxicity. Similarly, for patient F, the IMRT plan mean doses to both kidneys exceeded the tolerance of 18 Gy. This plan would not be clinically acceptable, thus patient F could not be treated with IMRT. Plans using IMPT with apertures, in contrast, had the lowest dose to the OARs and the lowest integral dose to the normal tissues for all patients. Both this study and other investigations demonstrated that IMPT with apertures is the preferred radiotherapy modalities and delivery techniques, especially for tumors that extend to a shallow depth.

The availability of proton therapy increases as more proton centers operate with IMPT as the chosen treatment planning and delivery technique. However, IMPT with apertures is currently not universally available to all the proton therapy centers for patient treatment. This is partly due to software (e.g., some treatment planning systems’ inability to support aperture‐based IMPT) or hardware (e.g., inability of the actual proton therapy gantry to support aperture‐based IMPT). It is unclear at this time whether software and hardware vendors are fully invested in IMPT with aperture functionality. If additional studies show results similar to this, suggesting an incremental benefit for aperture‐based IMPT for other tumor sites, it may encourage further research and development.

## 5 Conclusion

In this study, treatment plans of chest wall Ewing sarcoma were compared among IMRT, 3DCPT, and IMPT of different beam spot sizes and with/without beam apertures. The comparisons showed that depending on the chest wall subregion, proton treatment has the potential to minimize pulmonary, cardiac, renal, and hepatic toxicity, as well as second malignancies. Treatment plan using the smaller beam spot with beam apertures provided the best combination of target coverage and OAR sparing.

## Conflict of interest

None.
